# Phase I dose-escalation study of the mTOR inhibitor sirolimus and the HDAC inhibitor vorinostat in patients with advanced malignancy

**DOI:** 10.18632/oncotarget.11750

**Published:** 2016-08-31

**Authors:** Haeseong Park, Ignacio Garrido-Laguna, Aung Naing, Siqing Fu, Gerald S. Falchook, Sarina A. Piha-Paul, Jennifer J. Wheler, David S. Hong, Apostolia M. Tsimberidou, Vivek Subbiah, Ralph G. Zinner, Ahmed O. Kaseb, Shreyaskumar Patel, Michelle A. Fanale, Vivianne M. Velez-Bravo, Funda Meric-Bernstam, Razelle Kurzrock, Filip Janku

**Affiliations:** ^1^ Department of Investigational Cancer Therapeutics (Phase I Clinical Trials Program), Division of Cancer Medicine, The University of Texas MD Anderson Cancer Center, Houston, TX, USA; ^2^ Department of Internal Medicine (Division of Oncology), Washington University School of Medicine, St. Louis, MO, USA; ^3^ Department of Internal Medicine (Division of Oncology), Huntsman Cancer Institute and University of Utah School of Medicine, Salt Lake City, UT, USA; ^4^ Sarah Cannon Research Institute at HealthONE, Denver, CO, USA; ^5^ Medical Oncology, Thomas Jefferson University and Jefferson University Hospitals, Philadelphia, PA, USA; ^6^ Department of Gastrointestinal Medical Oncology, The University of Texas MD Anderson Cancer Center, Houston, TX, USA; ^7^ Department of Sarcoma Medical Oncology, The University of Texas MD Anderson Cancer Center, Houston, TX, USA; ^8^ Department of Lymphoma and Myeloma, The University of Texas MD Anderson Cancer Center, Houston, TX, USA; ^9^ Center for Personalized Cancer Therapy, University of California San Diego Moores Cancer Center, San Diego, CA, USA

**Keywords:** phase I, sirolimus, vorinostat, mTOR, HDAC

## Abstract

Preclinical models suggest that histone deacetylase (HDAC) and mammalian target of rapamycin (mTOR) inhibitors have synergistic anticancer activity. We designed a phase I study to determine the safety, maximum tolerated dose (MTD), recommended phase II dose (RP2D), and dose-limiting toxicities (DLTs) of combined mTOR inhibitor sirolimus (1 mg-5 mg PO daily) and HDAC inhibitor vorinostat (100 mg-400 mg PO daily) in patients with advanced cancer. Seventy patients were enrolled and 46 (66%) were evaluable for DLT assessment since they completed cycle 1 without dose modification unless they had DLT. DLTs comprised grade 4 thrombocytopenia (*n* = 6) and grade 3 mucositis (*n* = 1). Sirolimus 4 mg and vorinostat 300 mg was declared RP2D because MTD with sirolimus 5 mg caused significant thrombocytopenia. The grade 3 and 4 drug-related toxic effects (including DLTs) were thrombocytopenia (31%), neutropenia (8%), anemia (7%), fatigue (3%), mucositis (1%), diarrhea (1%), and hyperglycemia (1%). Of the 70 patients, 35 (50%) required dose interruption or modification and 61 were evaluable for response. Partial responses were observed in refractory Hodgkin lymphoma (−78%) and perivascular epithelioid tumor (−54%), and stable disease in hepatocellular carcinoma and fibromyxoid sarcoma. In conclusion, the combination of sirolimus and vorinostat was feasible, with thrombocytopenia as the main DLT. Preliminary anticancer activity was observed in patients with refractory Hodgkin lymphoma, perivascular epithelioid tumor, and hepatocellular carcinoma.

## INTRODUCTION

Phosphoinositide 3-kinase (PI3K)/Akt signaling is an important cell survival pathway [1]. Activation of the PI3K/Akt pathway is associated with uncontrolled cell proliferation and resistance to apoptosis [2]. Mammalian target of rapamycin (mTOR) is phosphorylated in response to PI3K/Akt activation. The PI3K/Akt/mTOR pathway is constitutively activated in many different human cancers, including ovarian, breast, and colon cancers and glioblastoma [3–5]. Several mTOR complex 1 (mTORC1) inhibitors are used in the clinic for the treatment of various cancers [6–8].

Sirolimus is an allosteric mTORC1 inhibitor that has immunosuppressive [9] and antitumor properties [10, 11]. It inhibits S6K and 4EBP1 phosphorylation, which decreases the translation of mRNAs that are critical for cell cycle progression, such as cyclin D1, and thus leads to cell cycle arrest and apoptosis [12]. In addition, sirolimus reduces the transcription of hypoxia-inducible factor (HIF1α) and subsequently leads to decreased production of vascular endothelial growth factor, demonstrating antiangiogenic effects in preclinical cancer models [13].

A paradoxical increase in p-AKT through disruption of a p70S6K-dependent negative feedback loop has been suggested as a mechanism of resistance to mTORC1 inhibition [14]. Developing targeting strategies that can abrogate p-Akt upregulation in response to mTORC1 inhibition is thus a putatively desirable approach for overcoming resistance to this family of drugs.

Post-translational modifications of chromatin histones are key regulators of gene expression [15]. These modifications include acetylation and deacetylation of lysines in the tails of the core histones controlled by the balanced action of histone deacetylases (HDACs) and histone acetyltransferases [16]. HDACs also target non-histone proteins, such as p53, tubulin, and transcription factors, and regulate cell proliferation, cell migration, and cell death. Aberrant expression of HDACs has been associated with diverse leukemias, lymphomas, and solid tumors [17]. Hydroxamate HDAC inhibitors, such as vorinostat, function as pan-HDAC inhibitors, targeting both class I and class II (including class IIb) HDACs. HDAC inhibitors kill cells through diverse mechanisms, including induction of oxidative injury, upregulation of death receptors, disruption of the cell cycle checkpoint, induction of heat shock protein 90 acetylation (leading to increased degradation of p-Akt), upregulation of proapoptotic proteins, and interference with proteasome function [18–20].

Vorinostat also may lead to inhibition of PI3K activity, possibly secondary to the modulation of vorinostat-induced gene expression [21]. Vorinostat diminishes the kinase activity of PIK3 *in vitro*, both in mantle cell lymphoma Jeko1 cells and in PC3 prostate cancer cells [21]. The combination of vorinostat and temsirolimus, an analogous ester of sirolimus, dramatically suppressed survivin levels and produced greater tumor growth inhibition and apoptosis than did single-agent temsirolimus *in vivo* [22]. Another preclinical study showed that, while HDAC inhibition alone led to inhibition of LKB1 and AMP-activated protein kinase and thus increased mTOR activity, the combination of an HDAC inhibitor and an mTOR inhibitor resulted in synergistic tumor cell death in Hodgkin lymphoma cell lines [23]. These preclinical data provided a mechanistic rationale for further exploration of this approach in clinical trials.

We hypothesized that combining vorinostat and sirolimus would increase the sensitivity of cancer cells to these drugs by simultaneously inhibiting mTOR, AKT, and HDAC. Therefore, we designed this study to determine the safety, maximum tolerated dose (MTD) and recommended phase II dose (RP2D), and dose-limiting toxicities (DLTs) of the combination of the mTOR inhibitor sirolimus (1 mg-5 mg PO daily, q 28 days) and the HDAC inhibitor vorinostat (100 mg-400 mg PO daily, q 28 days) in patients with advanced cancer.

## RESULTS

### Patient characteristics

**Figure 1:**
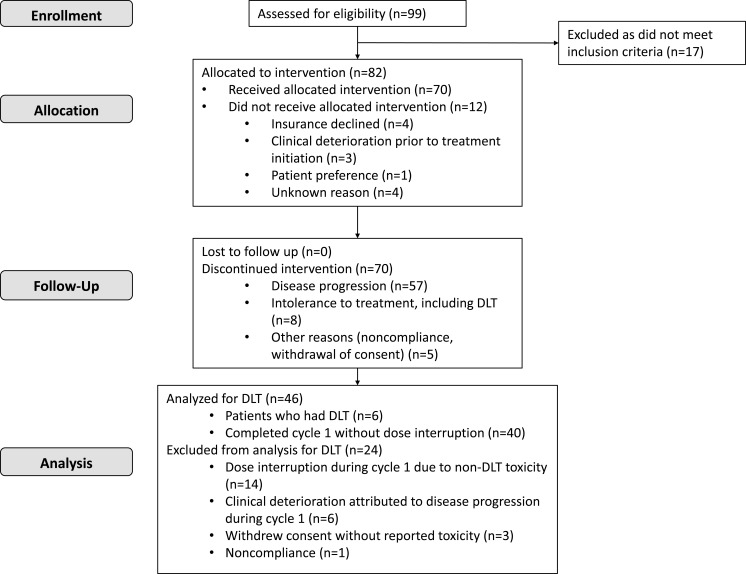
The CONSORT flow diagram depicting patients' identification, enrollment and treatment

From March 2010 to December 2012, a total of 99 patients were screened. Of those, 82 met eligibility criteria, and 70 were started on treatment at the dose escalation phase (Figure 1). For the 12 patients who did not start treatment, the reasons included lack of insurance coverage (*n* = 4), clinical deterioration (*n* = 3), patient preference (*n* = 1), or unknown reasons (*n* = 4). The 70 patients' demographic and clinical characteristics are shown in Table 1. There were 35 men and 35 women. Fifty-three (76%) patients were white, and the median age at study enrollment was 58 years (range, 16-79 years). Colorectal cancer, sarcoma, melanoma, and hepatocellular carcinoma comprised nearly half of the cases. The median number of treatment cycles on the protocol was 2 (range, < 1-20), and the median number of previous treatments was 4 (range, 0-9). Fifty-seven patients discontinued therapy because of disease progression, 8 because of intolerance, and 5 for other reasons, including noncompliance and withdrawal of consent.

**Table 1:** Demographic and clinical characteristics of patients with advanced cancer in a phase I study of sirolimus and vorinostat

Characteristic	Result
Sex, N (%)	
Female	35 (50%)
Male	35 (50%)
Median age at study enrollment, years (range)	58 (16–79)
Race, N (%)	
White	53 (76%)
Black	11 (16%)
Other	6 (9%)
Disease type, N (%)	
Colorectal cancer	11 (16%)
Sarcoma	9 (13%)
Melanoma	8 (11%)
Hepatocellular carcinoma	6 (9%)
Neuroendocrine carcinoma	5 (7%)
Non-small cell lung cancer	4 (6%)
Kidney cancer	3 (4%)
Thyroid cancer	3 (4%)
Appendiceal cancer	2 (3%)
Bladder cancer	2 (3%)
Endometrial cancer	2 (3%)
Hodgkin lymphoma	2 (3%)
Mesothelioma	2 (3%)
Ovarian cancer	2 (3%)
Pancreatic or ampullary carcinoma	2 (3%)
Salivary gland cancer	2 (3%)
Breast cancer	1 (1%)
Cervical cancer	1 (1%)
Gastric cancer	1 (1%)
Prostate cancer	1 (1%)
Carcinoma of unknown primary	1 (1%)
Number of treatment cycles, median (range)	2 (<1–20)
Number of prior therapies, median (range)	4 (0–9)

### Toxicity

All 70 patients were evaluated for toxicity, and 46 patients (66%) were evaluable for DLTs (patients who either had a DLT or completed cycle 1 without dose interruption). Dose level 5A, 5 mg of sirolimus and 300 mg of vorinostat, was declared the MTD of this combination on the basis of the DLTs; however, because of significant non-dose-limiting hematological toxicity, mainly prolonged or recurrent thrombocytopenia, 4 mg of sirolimus and 300 mg of vorinostat were established as RP2D for further investigation. Six patients experienced a DLT during the first cycle, including 5 with grade 4 thrombocytopenia and 1 with grade 3 mucositis (Table 2). One patient with grade 4 thrombocytopenia was treated at dose level 0; however, this patient had a baseline platelet count of 63,000/μL. The other DLTs were observed at either dose level 5A (*n* = 1) or dose level 6 (*n* = 4).

**Table 2:** Dose levels and DLTs in a phase I study of sirolimus and vorinostat in patients with advanced cancer

Dose level	Sirolimus (orally, mg daily)	Vorinostat (orally, mg daily)	Number of patients treated	Number of patients experiencing DLT
0	1	100	10	1 (G4 thrombocytopenia)
1	2	100	11	0
2	2	200	9	0
3	3	200	8	0
4	3	300	8	0
5	4	300	9	0
5A	5	300	6	1 (G4 thrombocytopenia)
6	4	400	9	4 (G4 thrombocytopenia, n=3; G3 mucositis, n=1)

DLT, dose-limiting toxic effect; G, grade.

Thrombocytopenia was the most common toxicity (Table 3): 20 patients experienced grade 3 (*n* = 12) or grade 4 (*n* = 8) thrombocytopenia during the study period, including those who experienced thrombocytopenia as a DLT during the first cycle. Thirteen patients experienced recurrent grade 2 or higher thrombocytopenia beyond the first cycle; 2 of these patients experienced prolonged grade 3 thrombocytopenia that required further dose modification and interruption. Other grade 3 and grade 4 toxicities included neutropenia (grade 3, *n* = 5; grade 4, *n* = 1), anemia (grade 3, *n* = 3; grade 4, *n* = 2), fatigue (grade 3, *n* = 2), diarrhea (grade 3, *n* = 1), and hyperglycemia (grade 3, *n* = 1). One patient had recurrent grade 4 anemia beyond the first cycle, and another had recurrent grade 4 neutropenia during the study. Thirty-five (50%) patients required dose interruptions and/or reduction. Of the 35, 6 patients had dose interruption due to DLT during the first cycle, 14 patients had other toxicity-related dose interruption during the DLT period, and 15 patients had dose interruptions after the first cycle (DLT window) was completed. Eight (11%) patients discontinued the study drugs because of intolerance (hematological toxicity, *n* = 4; nausea/vomiting or indigestion, *n* = 2; infection, *n* = 1, and renal insufficiency, *n* = 1).

**Table 3:** All grade 3 or grade 4 toxicities reported in a phase I study of sirolimus and vorinostat in patients with advanced cancer

Toxicity	G	*N* (%)
Thrombocytopenia	3	12 (17%)
	4	8 (11%)
Neutropenia	3	5 (7%)
	4	1 (1%)
Anemia	3	3 (4%)
	4	2 (3%)
Fatigue	3	2 (3%)
Diarrhea	3	1 (1%)
Hyperglycemia	3	1 (1%)
Mucositis	3	1 (1%)

G, grade.

### Efficacy

**Figure 2:**
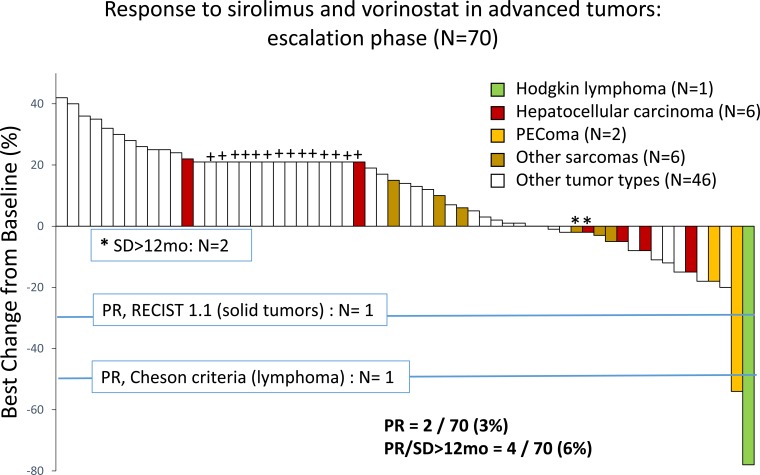
Waterfall plot depicts percentage change in target lesions in 70 patients with advanced cancer treated with sirolimus and vorinostat in the escalation phase Patients who either experienced clinical progression prior to first restaging images or had at least 1 restaging imaging study were considered evaluable for response (*n* = 61). Nine patients discontinued therapy for toxicity prior to first restaging and were excluded from the response evaluation. Those who discontinued therapy because of clinical disease progression, prior to restaging scans, were included (depicted arbitrarily as +20% and shown with +).

**Figure 3:**
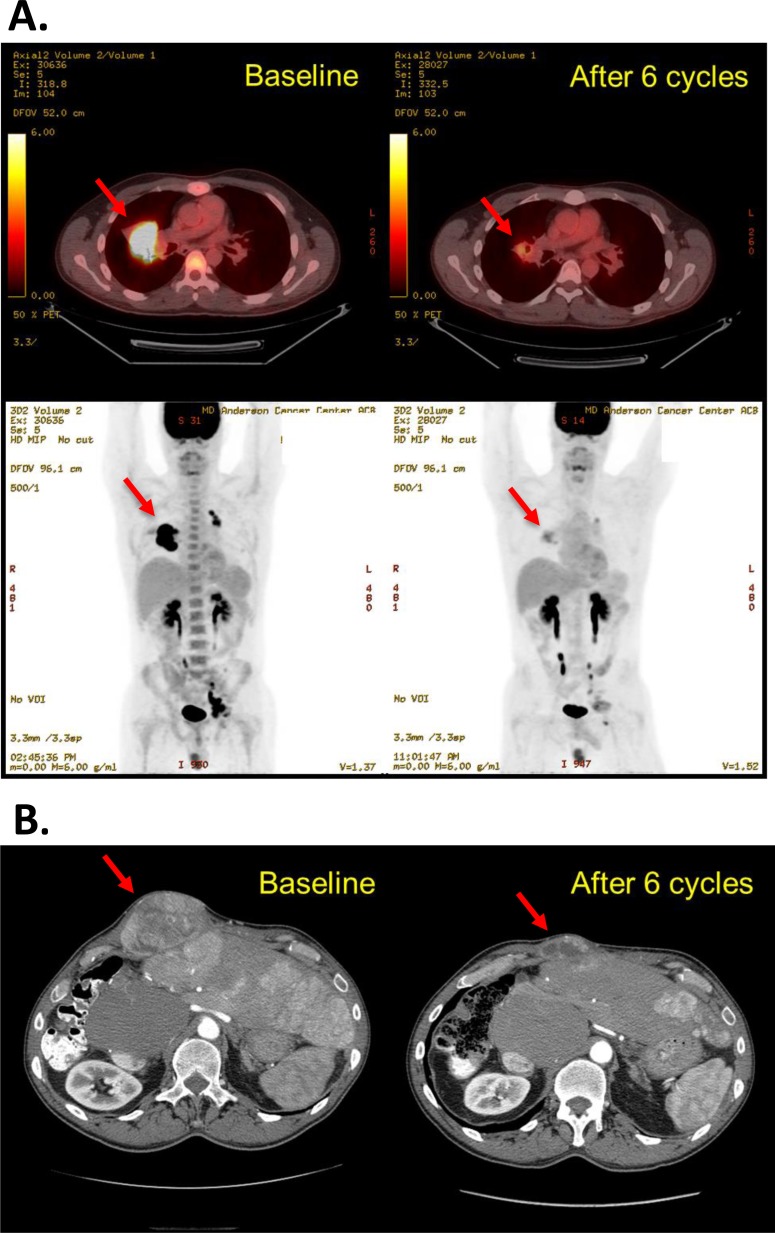
Representative restaging images of 2 patients who had a favorable response to therapy **A.** Patient with refractory relapsed Hodgkin lymphoma at baseline and after 6 cycles of treatment at dose level 5A. The best response by Cheson criteria was a partial response (−78% tumor size compared to baseline). He remained on treatment for 20 cycles. **B.** Patient with a perivascular epithelioid tumor at baseline and after 6 cycles of treatment at dose level 6. The best response by RECIST 1.1 was a partial response (−54% tumor size compared to baseline). He remained on treatment for 8 cycles.

Of the 70 treated patients, 14 experienced clinical progression before the first restaging scans and 47 underwent at least 1 restaging imaging procedure during treatment on the protocol, and thus 61 were considered evaluable for response. Four patients had a partial response (PR, *n* = 2) or durable stable disease for more than 12 months (SD>12months, *n* = 2), as shown in Figure 2. The objective response rate was 3%, 2 patients experiencing a PR during the escalation phase. One patient with relapsed, refractory Hodgkin lymphoma, who had had 8 lines of previous therapy, including an autologous stem cell transplant, experienced a 78% reduction in target tumor lesions per Cheson criteria after 6 cycles of therapy and remained on the protocol for 20 cycles (18.7 months), at which point he developed progressive disease. This patient was treated at dose level 5A. Another patient with perivascular epithelioid tumor, who was treated at dose level 6, had a gradual reduction by as much as 54% in sum of target lesions after 6 cycles of therapy and stayed on treatment for 8 cycles. One patient with hepatocellular carcinoma and 1 patient with fibromyxoid sarcoma had SD>12months. Representative imaging studies for these patients are shown in Figure 3. At the time of analysis, 57 (81%) patients had discontinued therapy because of disease progression. Other reasons for study discontinuation included toxicity (*n* = 8 [11%]), withdrawal of consent without reported study-related toxicities (*n* = 3 [4%]), and noncompliance (*n* = 2 [3%]). The median duration of follow-up was 27.3 weeks (range, 2.9-188 weeks). The median PFS was 9 weeks (95% CI: 8-10.3 weeks). At the time of analysis, 63 (90%) patients had died. The median OS was 24.3 weeks (95% CI: 19.1-31 weeks).

### Pharmacokinetics

Sirolimus levels were determined for 5 patients. Three of these patients were treated at dose level 5A (MTD), 1 at dose level 3, and 1 at dose level 6. Sirolimus levels during therapy ranged between 3.2 ng/mL and 33.5 ng/mL, with a mean of 20.5 ng/mL, which was above the therapeutic range recommended for post-transplantation immunosuppression in these patients. Among those who had sirolimus levels drawn, 2 patients had SD>12 months and 1 patient had a PR. All patients experienced grade 3 thrombocytopenia. Although the number of patients is small, no significant relationship between sirolimus level and degree of toxicity was observed on the basis of these patients' samples.

### Molecular profiles

Among the 70 patients treated, 48 had molecular testing of their tumor, and 41 of them were evaluable for disease response assessment (Supplementary Table 1); however, only 10 patients had comprehensive targeted next-generation sequencing for molecular alterations of at least 45 or more genes. Among the 4 patients who had PR or SD>12 months, 1 patient with Hodgkin lymphoma had *TSC2* loss, a molecular aberration that putatively activates the mTOR pathway. The molecular aberrations in patients who experienced a PR or SD>12 months are shown in Table 4. Complete list of detected molecular aberrations and treatment outcomes is in Supplementary Table 1.

**Table 4:** Molecular aberrations observed in patients who derived clinical benefit from the combination of sirolimus and vorinostat in a phase I study

Patient	Disease	Molecular aberration	Best response
1	Perivascular epithelioid tumor	*KIT* M541L*	PR
2	Hodgkin lymphoma	*TSC2* loss *XPO1* E571K	PR
3	Fibromyxoid sarcoma	*APC* A2122_C2123insA	SD>12 months
4	Hepatocellular carcinoma	*KIT* M541L*	SD>12 months

PR, partial response; SD, stable disease.

* Possible germline polymorphism.

## DISCUSSION

Our study demonstrated that the combination of 4 mg of sirolimus and 300 mg of vorinostat was feasible in patients with advanced malignancy. The DLTs included thrombocytopenia (5/6, 83%) and mucositis (1/6, 17%). Thrombocytopenia was the most frequent grade 3 or grade 4 treatment-related toxicity (28.5%). This is not surprising since both single-agent sirolimus and single-agent vorinostat can cause thrombocytopenia. For instance, sirolimus was associated with thrombocytopenia in up to 30% of patients who received higher doses (5 mg daily) after transplantation and in 10% of patients with advanced cancer [11, 24, 25]. Vorinostat was associated with grade 3 or grade 4 thrombocytopenia in 5%-20% of cases [26–29].

A similar combination of everolimus, an mTOR inhibitor, and panobinostat, a pan-HDAC inhibitor, was evaluated in a phase I clinical trial in patients with lymphoma, including Hodgkin lymphoma [30]. Patients enrolled in this study had similar toxicity profiles to those in our study, with a high incidence of thrombocytopenia (59%) and neutropenia (up to 50%) at the effective dose level. The DLT was grade 4 thrombocytopenia. Neutropenia was not as common in our study, which may be explained by the different patient populations: unlike some patients in the other study, many of the patients in the current dose-escalation study had a solid tumor, with no history of heavy bone marrow-suppressive therapy. The incidence of pneumonia was also higher with the panobinostat and everolimus combination; we did not observe this in our study with sirolimus and vorinostat.

In the current study, the 2 patients with Hodgkin lymphoma and 4 of the 6 patients with hepatocellular carcinoma experienced grade 3 or grade 4 thrombocytopenia during treatment. On the basis of our observations, we speculate that the patients who had undergone prior myelotoxic chemotherapy or had a decreased liver function reserve had more severe thrombocytopenia. It is notable that the degree of thrombocytopenia was often not cumulative, and many patients who experienced grade 3 or grade 4 thrombocytopenia had a spontaneous recovery, which even allowed dose reescalation of study drugs. Considering that the population included in our study had undergone multiple cancer treatments prior to study participation and that the inclusion criteria allowed baseline platelet counts of 50,000/μL, we believe that the severity of thrombocytopenia observed in this study was reasonably safe.

One patient in our study experienced grade 3 mucositis, which was more likely related to sirolimus than to vorinostat. Another 4 patients experienced grade 1 mucositis. This frequency was lower than expected considering that 60% of patients in the panobinostat and everolimus study had grade 1 to grade 2 mucositis during treatment [30]. We observed very few metabolic adverse events, such as hypertriglyceridemia or hyperglycemia, which are often associated with mTOR inhibitors. One patient did experience grade 3 hyperglycemia; this patient had had type II diabetes for more than 5 years prior to starting treatment on this protocol, along with other metabolic risk factors, including morbid obesity, hypertension, and hyperlipidemia.

We did not observe any strong correlation between molecular aberrations related to PI3K/Akt/mTOR pathway activation and response to therapy, although the generalizability of this observation may be limited because of the small number of patients. Two patients were found to have activating *PIK3CA* mutations. One patient had bladder cancer with *PIK3CA* H1047L and was treated at dose level 4; however, he discontinued therapy because of worsening renal function (> grade 3) and was not evaluable for response. Another patient had hepatocellular carcinoma with *PIK3CA* H1047R and was treated at dose level 6. He had SD, with a 15% decrease in tumor measurements after the second cycle, but the disease had progressed rapidly at the next restaging. Only 1 of the 4 patients who had a PR or SD>12 months had a molecular aberration related to the PI3K/Akt/mTOR pathway, a *TSC2* loss. Inactivation of *TSC2* leads to activation of mTOR; thus, it can be a target for mTOR inhibitor therapy. Preclinical and anecdotal evidence suggests that the loss of *TSC2* signal may predict a better response to mTOR inhibitor therapy [31, 32]. One patient with a PR and one patient with a SD> 12 months had *KIT* M541L mutation; however, previous data suggested that this might be a common germline polymorphism rather than a driver mutation [33].

The most remarkable response was seen in a patient with heavily pretreated Hodgkin lymphoma, which is consistent with the results of *in vitro* preclinical experiments [23]. There is *in vitro* evidence of the immunomodulatory effects of HDAC inhibitors, including suppression of T cell PD-1 expression [34], upregulation of PD-1 ligands [35], and inhibition of regulatory T cells, which have the potential to enhance the antitumor immune response [36]. In addition, inhibition of the PI3K/Akt/mTOR pathway has been shown to selectively reduce the activity of regulatory T cells [37]. Even though mTOR inhibitors in general are deemed to have immunosuppressive properties, we believe that in the context of this study the combination of HDAC and mTOR inhibition can in fact activate an anticancer immune response through its effects on the microenvironment as well as its direct inhibitory effect on cancer cells. Clinical response to immunotherapy with a checkpoint inhibitor has been remarkable in patients with Hodgkin lymphoma [38]. It is plausible that patients with tumor types that exhibit a higher degree of immune dysregulation and an inflammatory microenvironment, such as Hodgkin lymphoma and hepatocellular carcinoma, experienced a more significant benefit from this combination because of the potential immunomodulatory role of this combination. Finally, about half of the patients required dose interruptions or dose reduction, which could plausibly alter the efficacy. However, our sample size was limited to demonstrate any dose-response relationship.

This study has several limitations. First, 24 (34%) of the 70 patients were not available for DLT assessment, as protocol-defined DLT assessment required completion of the first cycle without dose reduction for any reason other than DLT. Some of these patients experienced clinical progression prior to completing the first cycle, and some had toxic effects that were significant enough to interrupt the doses but did not last long enough to meet the criteria for DLT. This may have resulted in underestimation of the toxic effects and thus identification of the RP2D at the lower dose level than the MTD. Second, both sirolimus and vorinostat are relatively infrequently used in the clinic because of the emergence of newer agents. Although our study findings suggest that combining mTOR and HDAC inhibitors can be meaningful, it might be challenging to find adequate support for further development because of the short residual patent life (if any) of both drugs in this combination. Nevertheless, the cost of this therapy is anticipated to decrease, which might be viewed favorably by cooperative groups or other non-commercial sources of funding. Furthermore, the protocol included correlative studies, but they were optional; at the time of analysis we did not have enough data to identify biomarkers of response. Finally, while most patients tolerated the treatment relatively well, some experienced dose-limiting or even dose-prohibitive thrombocytopenia.

In summary, combining mTORC1 inhibition with HDAC inhibition appears to be a safe and efficacious strategy for several cancer types. On the basis of the activity signals observed in this study, we are currently enrolling patients with Hodgkin and non-Hodgkin lymphoma, hepatocellular carcinoma, or perivascular epithelioid tumor into expansion cohorts.

## MATERIALS AND METHODS

This study was a non-randomized, open-label, dose-escalation phase I clinical trial of sirolimus and vorinostat (NCT01087554) performed at The University of Texas MD Anderson Cancer Center (MD Anderson). The primary objective was to determine the safety, MTD and RP2D, and DLTs of the combination of sirolimus and vorinostat in patients with advanced cancer. The protocol was approved by the Institutional Review Board. All participants gave informed consent prior to entering the study.

We enrolled patients with histologically confirmed metastatic or locally advanced cancer who were treated at the Clinical Center for Targeted Therapy at MD Anderson between March 2010 and December 2012. Among those who presented to the Clinical Center for Targeted Therapy, patients who met all the eligibility criteria were selected. All patients had disease that had failed to respond to standard therapy, had progressed despite standard therapy, or for which there was no available therapy that would prolong survival by at least 3 months. Patients were required to be off systemic therapy for at least 3 weeks before starting the protocol (or 5 half-lives in the case of biologics or targeted agents). Other inclusion criteria included an Eastern Cooperative Oncology Group performance status of 0, 1, 2, or 3; adequate organ and bone marrow function, as defined by an absolute neutrophil count of 1,000/μL or greater, a platelet count of 50,000/μL or greater, a total bilirubin level less than twice the institutional upper limits of normal (in the absence of Gilbert syndrome), an alanine aminotransferase level less than 2.5 times (less than 5 times for patients with liver involvement) the institutional upper limits of normal, and a creatinine level of 2.0 mg/dL or less; and the use of contraception during the study period. Patients were excluded if they had a history of myocardial infarction within 3 months prior to starting treatment, were pregnant or breastfeeding, or had undergone a major surgical procedure within 28 days before starting therapy. Palliative radiation therapy was allowed during the study treatment. The use of other standard or investigational anticancer agents was not allowed.

Patients were enrolled at 8 dose levels. The starting doses were 1 mg of oral sirolimus daily and 100 mg of oral vorinostat daily for 28 days, and doses were increased according to a standard “3+3” dose escalation design (Table 2). The study allowed to enroll up to 3 additional patients to each dose level to further evaluate safety. The concomitant use of CYP3A4 inhibitors was discouraged. Dose modification was allowed for treatment-related toxic effects. Patients continued treatment until they experienced disease progression or intolerable toxic effects or until the treating physician or patient felt that it was not in the patient's best interest to continue for any reason.

Patients who had DLT or completed cycle 1 without dose modification were evaluable for the DLT analysis. Patients not evaluable for DLT were replaced. The MTD was defined as the highest dose at which no more than 33% of patients developed a DLT. DLTs were treatment-related grade 3 or grade 4 non-hematologic toxic effects, as defined by the U.S. National Cancer Institute's Common Terminology Criteria for Adverse Events v 3.0, or as grade 4 hematologic toxic effects lasting more than 7 days or accompanied by fever (for neutropenia) or bleeding (for thrombocytopenia) during cycle 1. Grade 3 toxicities such as hyperglycemia, nausea, vomiting, diarrhea, rash, and asymptomatic lipase elevation were excluded from the DLTs if manageable with appropriate medication. Serum sirolimus levels were measured when available to assess pharmacokinetics. Vorinostat levels were not assessed. Tumor genomic analyses were performed whenever feasible using PCR or next-generation sequencing-based methods such FoundationOne or FoundationOne Heme (Foundation Medicine, Boston, MA), IonTorrent (Life Technologies, Carlsbad, CA), MassARRAY (Sequenom, San Diego, CA) and PCR-based DNA sequencing method that used primers designed by MD Anderson's Molecular Diagnostic Laboratory.

Response to therapy was assessed according to the Response Evaluation Criteria in Solid Tumors (RECIST) 1.1 [39] or Revised Response Criteria for Malignant Lymphoma (Cheson criteria) [40]. The median progression-free survival (PFS) and overall survival (OS) were calculated using the Kaplan-Meier method.

## SUPPLEMENTARY MATERIAL TABLE


