# Supplementary material for: Phase I dose-escalation study of the mTOR inhibitor sirolimus and the HDAC inhibitor vorinostat in patients with advanced malignancy
Source: Oncotarget. 2016 Aug 31;7(41):67521–31. doi: 10.18632/oncotarget.11750 (PMC5341894; doi:10.18632/oncotarget.11750)
Supplement: Supplementary file 1 [file oncotarget-07-67521-s001.pdf]

Phase I dose-escalation study of the mTOR inhibitor sirolimus and the HDAC inhibitor vorinostat in patients with advanced malignancy

Supplementary Material

Supplementary Table 1. Detected molecular aberrations, affected pathways and treatment outcomes

Molecular Aberration	Affected pathway	PR	SD > 12 months	SD < 12 months	PD	Not evaluable for response
APC A2122_C2123insA	Wnt		1			
BRAF V600E	MAPK				1	
GNAQ Q209P	MAPK				1	
KIT M541L[1], *KDR* C482R[1]	KIT, VEGFR			1		
KIT M541L[1]	KIT	1	1			
KRAS G12C	MAPK			1		
KRAS G12D	MAPK				1	
KRAS G12S	MAPK					1
KRAS G13D	MAPK			1		
MET N375S	MET			1		
NF1 R1241*, *BAP1* truncation, *CTNNB1* N387K	MAPK, BAP, Wnt				1	
NRAS Q61K	MAPK				1	
NRAS Q61R	MAPK			1	1	1
PIK3CA H1047L	PI3K					1
PIK3CA H1047R, *CTNNB1* S37C, *PTPRD* S1845fs*2	PI3K, Wnt, STAT3			1		
PTEN R335*, *CDKN2A/B* loss	PI3K, CDK			1		
TP53 G245S	TP53					1
TP53 L194F	TP53			1		
TP53 R175H	TP53				2	
TSC2 loss, *XPO1* E571K	mTOR, XPO1	1				
No aberration				8	13	3
Not done				7	14	2

Abbreviations: PR, partial response; SD, stable disease; PD, progressive disease
[1]Possible germline polymorphism
